# Tripartite species interaction: eukaryotic hosts suffer more from phage susceptible than from phage resistant bacteria

**DOI:** 10.1186/s12862-017-0930-2

**Published:** 2017-04-11

**Authors:** Carolin C. Wendling, Agnes Piecyk, Dominik Refardt, Cynthia Chibani, Robert Hertel, Heiko Liesegang, Boyke Bunk, Jörg Overmann, Olivia Roth

**Affiliations:** 1 GEOMAR, Helmholtz Centre for Ocean Research, Evolutionary Ecology of Marine Fishes, Düsternbrooker Weg 20, 24105 Kiel, Germany; 2 Present address: Max Planck Institute for Evolutionary Biology, Department of Evolutionary Ecology, August-Thienemann-Straße 2, 24306 Plön, Germany; 3 Institute of Natural Resource Sciences, Zurich University of Applied Sciences, School of Life Sciences and Facility Management, Campus Grüental, CH-8820 Wädenswil, Switzerland; 4 Institute for Microbiology and Genetics, Georg-August University Goettingen, Grisebachstr. 8, 37077 Goettingen, Germany; 5 Leibniz Institute DSMZ-German Collection of Microorganisms and Cell Cultures, Inhoffenstr. 7B, 38124 Braunschweig, Germany

**Keywords:** Temperate phages, Prophages, Bacteria-phage infection network, *Vibrio*, Tripartite interaction

## Abstract

**Background:**

Evolutionary shifts in bacterial virulence are often associated with a third biological player, for instance temperate phages, that can act as hyperparasites. By integrating as prophages into the bacterial genome they can contribute accessory genes, which can enhance the fitness of their prokaryotic carrier (lysogenic conversion). Hyperparasitic influence in tripartite biotic interactions has so far been largely neglected in empirical host-parasite studies due to their inherent complexity. Here we experimentally address whether bacterial resistance to phages and bacterial harm to eukaryotic hosts is linked using a natural tri-partite system with bacteria of the genus *Vibrio*, temperate vibriophages and the pipefish *Syngnathus typhle*. We induced prophages from all bacterial isolates and constructed a three-fold replicated, fully reciprocal 75 × 75 phage-bacteria infection matrix.

**Results:**

According to their resistance to phages, bacteria could be grouped into three distinct categories: highly susceptible (HS-bacteria), intermediate susceptible (IS-bacteria), and resistant (R-bacteria). We experimentally challenged pipefish with three selected bacterial isolates from each of the three categories and determined the amount of viable *Vibrio* counts from infected pipefish and the expression of pipefish immune genes. While the amount of viable *Vibrio* counts did not differ between bacterial groups, we observed a significant difference in relative gene expression between pipefish infected with phage susceptible and phage resistant bacteria.

**Conclusion:**

These findings suggest that bacteria with a phage-susceptible phenotype are more harmful against a eukaryotic host, and support the importance of hyperparasitism and the need for an integrative view across more than two levels when studying host-parasite evolution.

**Electronic supplementary material:**

The online version of this article (doi:10.1186/s12862-017-0930-2) contains supplementary material, which is available to authorized users.

## Background

Infection of parasites by other parasites (i.e. hyperparasitism) plays an important role in the evolution of hosts and parasites. Micro-hyperparasites, for instance bacteriophages, are fundamental in determining the outcome of bacterial diseases [1]. To understand the ecology and evolution of bacterial diseases, it is necessary to extend the view of dual species interactions to tripartite interactions where the phage, its bacterial carrier and a eukaryotic host are involved. Such tripartite interactions have been well studied in systems using lytic phages, of which many demonstrate a trade-off between phage resistance and bacterial virulence (for a recent review see [2]). However, patterns of resistance and virulence between temperate phages, their bacterial carriers and the eukaryotic hosts are largely unexplored.

In contrast to lytic phages, temperate phages have two transmission modes. After infecting a bacterium they can either be transmitted horizontally through cell lysis (lytic cycle), or vertically as prophages, whereby the phage genome is integrated into the bacterial chromosome (lysogenic cycle). Indeed, prophages constitute up to 20% of the bacterial genome and are major contributors to the large genomic and phenotypic variation among bacterial strains of the same species [3].

During lysogeny the fitness of the prophage and its bacterial carrier is aligned, which explains instances where prophages protect their hosts against superinfection [4] or provide them with genes that increase bacterial proliferation [3]. However, prophages have also been described as molecular time bombs [5] that, either spontaneously or in response to specific environmental conditions, kill their carriers through cell lysis and switch back to the lytic cycle [3, 5].

While bacteria are in constant coevolutionary interaction with their eukaryotic host, they simultaneously face selection by their micro-hyperparasites, i.e. lytic phages. For instance, evolution of resistance in *Pseudomonas aeruginosa* to lytic ΦPP/and ΦE79 resulted in an upregulation of virulence genes, which ultimately increased virulence against mammalian cells [6]. In contrast, resistance against lytic phages in *Flavobacterium columnare* reduced bacteria gliding motility and thus virulence against its eukaryotic host [7].

We here aimed to extend the existing knowledge of micro-hyperparasitism in phage – bacteria – eukaryotic host interactions using temperate phages. Specifically, the objective of the present study is to investigate resistance patterns to temperate phages in a natural temperate phage – bacterial interaction and its relationship to bacterial harm in an animal host. By using bacteria of the genus *Vibrio*, their derived prophages, and one of their eukaryotic hosts, the broad-nosed pipefish *Syngnathus typhle* as a model system, we addressed the question if bacterial resistance to temperate phages and bacterial harm to eukaryotic hosts can be linked.

While in a variety of human pathogenic strains, *Vibrio* virulence can be directly linked to the presence of prophage [8–10], we lack insight that goes beyond the knowledge about human pathogenic strains and addresses *Vibrio*-phage interactions covering a broader range of environmental isolates. Here, we present experimental data on the interaction between 75 environmental *Vibrio* isolates and their associated prophages as well as on the impact of a subset of these bacterial isolates to the natural eukaryotic host, the pipefish. We conducted fully reciprocal cross-infections between all bacteria and their derived phage lysates, and experimentally challenged pipefish with nine of the isolates that differed in phage resistance. Based on the relative gene expression of pipefish immune genes, we observed that phage resistant bacteria are less harmful than phage susceptible bacteria. Our results indicate that bacteria with a phage-susceptible phenotype are more virulent against their eukaryotic host and suggest that temperate phages are important in shaping bacterial virulence in the marine realm.

## Methods

All *Vibrio* strains used in the present study had been isolated from nine healthy broad-nosed pipefish *Syngnathus typhle* collected in the Kiel Fjord during a previous study [11]. Labels were given according to the sampling area (first letter ‘K’ refers to the study site: Kiel), the fish individual (first number), the organ (second letter: ‘E’ referring to eggs, ‘K’ referring to gills, and ‘M’ referring to the whole intestines) and *Vibrio* colony number (second number). Healthy pipefish harbour a highly diverse community of bacteria of the genus *Vibrio spp*. that show a strong spatial diversification across Europe [11]. While most *Vibrio* are harmless, some are responsible for major disease outbreaks. For instance, several members of the *V. alginolyticus* and *V. splendidus* clade have been isolated from seahorses with signs of infections [12], while *V. harveyi* causes almost 90% mass mortalities in captive bred seahorses [13].

### *Vibrio* phylogeny

To determine the genetic affiliation of each *Vibrio* isolate we used a multi locus sequence analysis (MLSA) approach based on partial DNA sequences of 3 different genes (16S rRNA, *recA* and *pyrH).* Bacterial DNA was isolated from cell pellets of overnight cultures using the DNeasy 96 Blood & Tissue Kit (*Qiagen*) according to the manufacturers protocol. Amplification followed previously established protocols [14]. Primer details are listed in supporting information (Additional file 1: Table S1). PCR products were purified using ExoSAP (*Fermentas*) with 0.4 μl FastAP, 0.2 μl ExoI and 1.4 μl H_2_O per 2 μl PCR Product. Sequences were obtained on an ABI 3130xl Genetic Analyser (*Applied Biosystems*) using standard Sanger sequencing with ABI BigDye Terminator v3.1 Cycle Sequencing kit (*Applied Biosystems*). The thermal program consisted of an initial denaturation step (60 s at 96 °C) followed by 25 cycles (10 s at 96 °C, 5 s at 55 °C, 5 min at 60 °C).

### Whole genome sequencing

DNA for sequencing was isolated from cultures grown in Medium101 (Medium101: 0.5% (w/v) peptone, 0.3% (w/v) meat extract, 3.0% (w/v) NaCl in MilliQ water). The cultures were grown 16 h at 25 °C 250 rpm. High molecular weight DNA was prepared using Qiagen Genomic Tip/100 G from Qiagen, Hilden, Germany. SMRTbell™ template library was prepared according to the instructions from PacificBiosciences, Menlo Park, CA, USA, following the Procedure & Checklist - 10 kb Template Preparation Using BluePippin™ Size-Selection System. Briefly, for preparation of 15 kb libraries 8 μg genomic DNA was sheared using g-tubes™ from Covaris, Woburn, MA, USA. DNA was end-repaired and ligated overnight to hairpin adapters applying components from the DNA/Polymerase Binding Kit P6 from Pacific Biosciences, Menlo Park, CA, USA. Reactions and BluePippin™ Size-Selection to 7 kb were performed according to the instructions of the manufacturer (Sage Science, Beverly, MA, USA). Conditions for annealing of sequencing primers and binding of polymerase to purified SMRTbell™ template were assessed with the Calculator in RS Remote, Pacific Biosciences, Menlo Park, CA, USA. SMRT sequencing was carried out on the PacBio *RSII* (Pacific Biosciences, Menlo Park, CA, USA) taking one 240-min movie for each SMRT cell using P6 chemistry. In total one SMRT cell per strain was run for eight selected *Vibrio alginolyticus* strains. Genome assembly was performed with the RS_HGAP_Assembly.3 protocol included in SMRT Portal version 2.3.0. The number of postfiltered reads and the average read length of the reads is summarized in Additional file 2: Table S5, as well as the number of contigs obtained after primary assembly. Each contig was trimmed and circularized to obtain the two typical *Vibrio* chromosomes as well as additional plasmids and artificial contigs were removed. Automated genome annotation was carried out using Prokka [15].

### Phage-bacteria cross infection assay

We used standard spot-assays to construct a three-fold replicated, fully reciprocal phage-bacteria infection matrix [16].

#### Prophage induction

All *Vibrio* isolates were induced with mitomycin C (Sigma) as described in [4] with some modifications: bacteria were grown in liquid Medium101 (Medium101: 0.5% (w/v) peptone, 0.3% (w/v) meat extract, 3.0% (w/v) NaCl in MilliQ water) at 250 rpm and 25 °C overnight. Cultures were diluted 1:100 in fresh medium and grown for another 2.5 h at 250 rpm and 25 °C to bring cultures into exponential growth before adding mitomycin C at a final concentration of 0.5 μg/ml. Samples were incubated in an automated plate reader (TECAN infinite M200) for 4 h at 25 °C and mixed periodically. Bacterial lysis upon prophage induction was monitored via optical density at 600 nm (measured every other minute). We determined bacterial lysis time at induction as the time at which turbidity of the culture peaks (for details see [4]). After 4 h, lysates were centrifuged at 6000 *g* for 15 min and the supernatant was ten-fold diluted in TM buffer (modified from [17]): 50% (v/v) 20 mM MgCl_2_, 50% (v/v) 50 mM Tris-HCl, pH 7.5).

#### Spot assay

To determine bacterial susceptibility to the different phage lysates we used standard spot assays, in which a lawn of host bacteria is grown in a medium overlaid on agar plates [16]. Phage lysates are spotted on the overlaid medium and may infect bacteria. Phage infection is visible as plaques, i.e. circular clear or turbid zones where a lytic infection has spread through the bacterial lawn. Overnight cultures of bacterial strains were diluted 1:10 in fresh medium and grown for 2 h before they were mixed with the overlay medium as follows: 200 μl of exponentially growing cells were added to 4 ml Medium101 soft agar (0.4%) at 41 °C. The medium was poured onto Petri dishes containing 20 ml Medium101 agar (1.5% (w/v)). After 30 min, 2 μl of each phage lysate were spotted onto the plates. Controls on every plate were 2 μl uninduced bacterial culture, Medium 101, Medium 101 with 0.5 μg/ml mitomycin C, and TM buffer. Plates were dried for 30 min before incubation at 25 °C for 20 h. Each bacterial strain was scored as either susceptible (plaque formation) or resistant (no plaque formation) to each of the phage lysates. Similarly, each phage lysate was scored as either infective (plaque formation) or non-infective (no plaque formation).

We are aware that plaque formation may be misinterpreted by thinning of the bacterial lawn, which can be associated with defective prophages [18], colicins [19] or other toxic components in the supernatant of mitomycin C treated cultures. To support that the supernatants do indeed contain phage particles, we used a serial dilution on a susceptible host ranging from 10^−1^ to 10^−8^ and only scored those isolates, where individual plaques were observed. In addition, we isolated viral DNA (MasterPure DNA Purification Kit, Epicentre) from the supernatants to perform a standard agarose gel electrophoresis with 0.8% agarose and a 1 kb GeneRuler (Fermentas) as marker. Based on these two approaches we could confirm that all mitomycin C treated culture supernatants contained viral particles, which have ssDNA genomes of ~6 kb.

### Infection experiment

As the majority of our bacterial isolates (71 out of 75) could be assigned to the *Vibrio alginolyticus* clade, all subsequent analyses as well as the infection experiment are based on the *V. alginolyticus* isolates only.

#### Experimental procedure

Out of the 71 *Vibrio alginolyticus* strains we selected three strains that were highly susceptible to prophages (further named HS-bacteria), three strains that were intermediate susceptible to prophages (further named IS-bacteria) and three strains that were resistant to prophage infection (further named R-bacteria).

Pregnant male pipefish were randomly caught from the Kiel Fjord in July 2014 and transported to our laboratory facility in Kiel, Germany. Male pipefish were kept separately in 80-L aquaria and fed twice a day with live and frozen mysids. Immediately after birth, fathers were removed from the aquaria and juveniles were fed twice a day with *Artemia salina* naupliae for another 3 weeks.

Selected bacteria were grown under agitation at 25 °C as described in [20]. After 24 h we adjusted the concentration of each strain to 5 × 10^8^ cells/ml according to [14]. Prior to the start of the infection experiment we pooled 36 fish from nine different pregnant males and injured the skin of each fish with a sterile needle. Afterwards fish were kept separately in small 50-ml beakers, which either contained 10^6^ cells/ml of each respective *Vibrio* isolate diluted in PBS or only PBS, which served as a control treatment. We infected nine fish per strain resulting in 108 fish in total. After 2 days all fish were killed with a lethal dose of MS222, immersed in RNA-later and stored at 4 °C until RNA-extraction. We considered 2 days as an optimal time point to end the experiment for two reasons: a) we wanted to give the immune system time to react to the infection, and b) we observed in previous studies that fish mortality during a controlled infection experiment starts on average 3 days after infection.

#### Gene expression

Expression of 44 target genes relative to two housekeeping genes was analysed using a Fluidigm BioMark™ as described in Beemelmanns and Roth [21]. Briefly, we used 22 target genes assigned to the innate immune system, three to the complement component system, seven target genes assigned to the adaptive immune system and 15 target genes assigned to gene silencing or activation through DNA and histone methylation/demethylation and histone acetylation/deacetylation. Details about function of genes, sequences and primer design can be found in [21] and are listed in Additional file 3: Table S2.

We extracted RNA from whole juvenile fish using an RNeasy 96 Universal Tissue Kit (Qiagen) according to the manufacturer’s protocol. RNA concentration was adjusted to a total of 800 ng/μl per sample and subsequently transcribed into cDNA using the Quanti Tect Reverse Transcription Kit (Qiagen), which includes a genomic DNA (gDNA) digestion. After pre-amplification (for details see [21], samples and primers (two technical replicates per gene) were filled into specific inlets into the 96.96 dynamic array IFC (GE-chip) and measured in the BioMark™ system applying the GE fast 96.96 PCR protocol according to Fluidigm instructions. We included non-template controls (NTC), controls for gDNA contamination (-RT) and standard samples for inter-run calibration.

#### Infection intensity

To estimate the amount of viable *Vibrio* counts within infected pipefish we determined infection intensity, i.e. colony forming units (CFU) by plating 2 μl of the whole fish-suspension (which has been produced for total RNA extraction) on *Vibrio* selective Thiosulfate Citrate Bile Sucrose Agar (TCBS) plates (Fluka Analytica). Plates were incubated at 25 °C for 24 h. Afterwards CFU were counted for each fish.

### Bacterial properties

#### Growth rate

We generated 24 h growth curves of all selected strains, to identify potential differences in growth rates between bacterial groups (HS, IS, R) that might result from increasing costs of phage resistance.

#### Twitching motility

We further determined bacterial twitching motility based on a standard motility assay to determine if resistance to phages can be assigned to pilus mutations. In brief, aliquots of equal number of cells were stab inoculated on petri dishes containing TCBS agar (Fluka Analytica) and incubated at 25 °C for 48 h. After incubation a hazy zone of growth at the interface between the agar and the polystyrene surface was observed and its surface area quantified using ImageJ. The surface area was calculated as follows: if the surface area is circular in shape, we used the formula 2rπ, where r = 1/2 the diameter. If the surface area is oval in shape, measures of the shortest and longest diameter were taken and the surface area calculated according to the formula, π x a x b, where a =1/2 the longest and b = 1/2 the shortest diameter.

### Statistical analysis

All statistics were performed in the R 3.1.2 statistical environment (R Foundation for statistical computing) unless otherwise stated.

#### Phylogenetic analysis

MLSA was performed as described in [14] with the following modifications: All sequences were manually edited and automatically assembled using CodonCode Aligner v3.7.1.2. Edited gene sequences were compared against published sequences in NCBI GenBank using BLASTN algorithm with default settings based on 99% sequence identity to assign *Vibrio* isolates to putative close phylogenetic relatives. After assembly and alignment of concatenated sequences (2507 bp) using MUSCLE [22], we constructed a phylogenetic tree using the Bayesian Markov chain Monte Carlo (MCMC) method as implemented in MrBayes version 3.2.5 [23, 24]. The generalised time reversible model plus invariant sites (GTR + I), as suggested by the Akaike information criterion (AIC) given by jModelTest [25], was used as statistical model for nucleotide substitution. The MCMC process was repeated for 10^6^ generations and sampled every 5000 generations. The first 2000 trees were deleted as burn-in processes and the consensus tree was constructed from the remaining trees. Convergence was assured via the standard deviation of split frequencies (<0.01) and the potential scale reduction factor (PSRF ~ 1). The resulting phylogenetic tree and associated posterior probabilities were illustrated using FigTree version 1.4.2 (http://tree.bio.ed.ac.uk/software/figtree/.

#### Whole genome analysis

We calculated the phylogenetic relationship between the eight fully sequenced *Vibrio* strains using a whole genome alignment phylogeny-based approach. The alignment was calculated using Mugsy [26], and only the relevant LCBs (local collinear blocks) aligned regions present in all analyzed strains were extracted using Phylomark. These regions were concatenated and positions with gaps removed [27]. A heuristic maximum-likelihood phylogenetic tree was calculated from the resulting core alignment (528,197 bp) using FastTree2 [28] and visualized using Interactive tree of life (iTOL) v3 [29]. We screened the sequenced genomes for selected common virulence factors, such as virulence islands and type 2 toxin-antitoxin system as wells as the presence of a CRISPR/Cas system and differences in methylation patterns. In detail, *Vibrio* Genomic islands were predicted using IslandViewer [30]. Type II TA modules were screened using TAfinder [31], a web-based tool to identify type II toxin-antitoxin (TA) loci in bacterial genomes. Potential toxin-like candidates were predicted using ClanTox [32]. SMRT sequencing data of all strains was mapped to the eight assembled genome sequences of *V. alginolyticus*, using the BLASR algorithm (Pubmed-ID 22988817) as implemented in Pacific Biosciences’ SMRT Portal 2.3.0 within the “RS_Modification_and_Motif_Analysis.1” protocol applying default parameter settings.

#### Network analysis

After confirmation that the three infection matrices were not significantly different from each other (Mantel test; Monte-Carlo test observation based on 9999 permutations > 0.085; *p* < 0.001) we calculated a consensus matrix, in which we considered an infection to be positive if plaque formation was visible in at least two of the three replicates. Subsequent network analysis was performed on the consensus matrix using the bipartite package [33] and the Falcon interactive Mode for R [34]. Nestedness was calculated using the NODF index, which estimates nestedness based on overlap and decreasing fill. We used the SS null model to test for significance of the nestedness score.

#### Gene expression

A detailed description of our gene expression analysis is given in [21]. In short, we calculated the mean cycle time (ct) for each of the two replicates. We used qbase^+^ (version 2.6.1 [35]) to calculate the optimal number of housekeeping genes and found that the combination of the two housekeeping genes ubiquitin (Ubi) and ribosome protein (Ribop) showed the highest stability (average geNorm M ≤ 0.5). After removal of samples with a coefficient of variation larger than 4% we calculated the geomean Ct of the two housekeeping genes to quantify the relative gene expression of each target gene by calculating - ∆Ct. We used a multivariate analysis of variance (MANOVA) using the Pillai’s trace statistics with - ∆Ct values as dependent variable and bacterial group as well as strain nested within bacterial group as the independent variable. MANOVA was followed up by univariate analyses of the single genes. We further conducted a principal component analysis (PCA) using the ade4 package [36] to assess clustering according to the bacterial groups based on differences in expression patterns.

#### Viable *Vibrio* counts

We analysed the amount of CFU using a Kruskal-Wallis test for non-parametric data.

#### Bacterial growth rate

We used a linear mixed effect model with a Maximum likelihood error distribution using lme (package nlme) with bacterial group (HS, IS, R), time as well as their interaction as fixed effect and strains as random effect.

#### Twitching motility

We used a linear model to estimate differences in twitching motility based on differences in surface areas using bacterial group as fixed variable.

#### Lysis time

We defined lysis time as the time at which turbidity of the culture peaks [4]. According to the infectivity pattern of the derived prophages we grouped bacterial strains into three categories (HI: High infectivity, II: Intermediate infectivity, NI: No infectivity). We estimated the effect of these three bacterial groups on lysis time using a linear model (function: lm) followed by Tukey’s HSD posthoc test (R-package lsmeans).

## Results

### *Vibrio* phylogeny

**Fig. 1:**
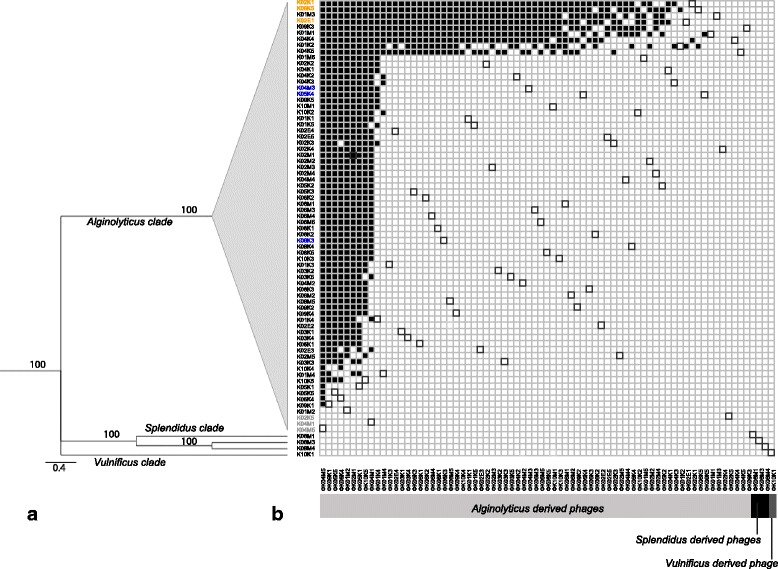
**a** Collapsed *Vibrio* phylogeny of the 75 isolates based on three concatenated housekeeping loci (16S rRNA *recA* and *pyrH*, 2507 bp) using the Bayesian Markov chain Monte Carlo (MCMC) method. The 75 isolates were separated into three major clades, i.e. *Alginolyticus* clade, *Splendidus* clade and *Vulnificus* clade. Nodes are labelled with posterior probabilities. **b** *Vibrio*/phage cross-infection matrix. Rows and columns represent bacteria and phage lysates. *Black cells* indicate infection success. Framed cells depict cross-inoculation between a lysogen and its derived phage lysate. Strains used in the infection experiment are highlighted in *orange* (HS-bacteria), *blue* (IS-bacteria) and *grey* (R-bacteria)

**Fig. 2:**
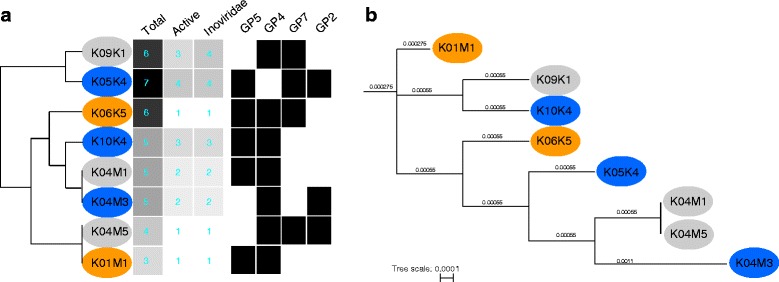
**a** Clustering of the eight sequenced strains based on a similarity matrix containing the total number of prophages (Total), the total number of active prophages (Active), the total number of inoviridae (Inoviridae) and presence (*black square*)/absence (white square) of shared prophages that have been found in more than one genome (Φ1, Φ2, Φ6, Φ18) different colours correspond to bacterial groups: orange: HS bacteria, blue: IS bacteria and R: resistant bacteria; **b** Maximum likelihood tree based on whole genome alignment of the eight sequenced strains

*Vibrio* phylogeny was constructed based on three concatenated housekeeping loci (16 s rRNA, *recA* and *pyrH*) representing 2,507 total nucleotides using the Bayesian Markov chain Monte Carlo (MCMC) method. The 75 isolates were separated into three major clusters, of which we could assign 71 strains to the *Alginolyticus* clade, three strains to the *Splendidus* clade and one strain to the *Vulnificus* clade (Fig. 1a). All strains belonging to the *Alginolyticus* clade had a 100% sequence identity based on the concatenated alignment and were therefore grouped by collapsing the internal branches within the *Alginolyticus* clade. However, based on a whole genome alignment of the selected eight strains, we could show that the isolates represent different strains (Additional file 4: Table S3 and Fig. 2b). These differences are mainly caused by different integrated prophages at different insertion sites, which might explain the observed distinct phenotypes.

### *Vibrio-*phage cross infection network

We found inducible prophages in all *Vibrio* isolates. In 64 out of the 71 *Alginolyticus* isolates single plaques were visible at dilutions of 10^−6^ to 10^−8^, however, they had fringed edges and were often overlapping and thus not clearly discernable making it impossible to count single PFUs. In addition, we could confirm the presence of prophages in the supernatants by DNA extraction and subsequent gel-electrophoresis, showing products of around ~6 kb, for all 71 isolates. A screening of the genomes of the complete sequenced strains confirmed the presence of several prophage loci within each of the genomes (Fig. 2a). The prophages include two that are shared by all strains (i.e. Φ1 and Φ2, Fig. 2a) as well as prophages which are unique within their encoding genome. On average less than half of all integrated prophages per strains are active of which the majority could be identified as *Inoviridae*. We found that R bacteria contain only one active prophage, while IS and HS bacteria contain on average two and three active prophages.

Based on all *Vibrio* strains and their induced prophages we generated a three-fold replicated 75 × 75 cross-infection matrix resulting in 16,875 inoculations. Among the 75 tested lysogens, 74 were homoimmune, i.e. immune to lytic infection by their own phage-lysates. The observed phage bacteria infection network (PBIN) is significantly nested: NODF nestedness score = 80.88; z-score = 126.63; *p* < 0.001 (Fig. 1b, for single matrices see Additional file 5: Figure S1). Overall, 15.98% of the phage-bacteria combinations resulted in lytic infection success, which corresponds to a network connectance of 0.16. We observed that infections occurred only within the strains of the *Alginolyticus* clade, while the non-*alginolyticus* isolates could not get infected by any of the phage lysates nor could their phage suspensions infect any of the *V. alginolyticus* strains. Therefore we excluded non-*alginolyticus* bacteria from the rest of the analysis and the infection experiment on juvenile pipefish.

Most of the bacteria (82%) were susceptible to 13% of the phage lysates (thereafter called intermediate-susceptible (IS) bacteria), while 13% of the bacteria were highly susceptible to the majority (77%) of the phage lysates (thereafter called HS-bacteria). Approximately 5% of the bacteria were resistant against all phage lysates (thereafter called R-bacteria) whereas 10% of the phage lysates were not able to cause a visible lytic infection using a standard spot assay. Bacteria from these three phenotypic groups do not cluster based on their genotype (Fig. 2b). All three bacterial groups contained bacteria from diverse organs of different fish. Infection patterns could therefore not directly be linked to within population differentiation.

**Fig. 3:**
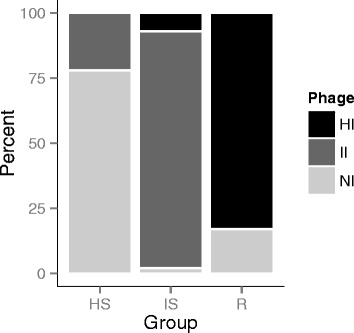
Relative proportion of high infective (HI; *black*), intermediate infective (II; *dark grey*) and non-infective (NI; *light grey*) phage lysates per bacterial group (HS highly susceptible, IS: intermediate susceptible and R: resistant)

Within bacteria and phage lysates from the *Alginolyticus* clade we detected a significant infection pattern: five out of nine phage lysates from HS-bacteria were non-infectious, while the remaining four could infect other strains, which themselves were exclusively highly susceptible. In contrast, most phage lysates derived from R bacteria (3 out of 4) could infect the majority of the 71 *V. alginolyticus* strains, while only one phage lysate could not cause a lytic infection on any of the tested strains (Fig. 3).

### Infection experiment

We used a controlled infection experiment on juvenile pipefish to directly test whether bacterial resistance to phages and bacterial harm to eukaryotic hosts can be linked. To control for clade effects all strains used in the infection experiment belonged to the *Alginolyticus* clade.

#### Viable *Vibrio* counts

Overall, the amount of CFU differed significantly between fish treated with PBS compared to fish infected with bacteria groups (Kruskal-Wallis test for non-parametric data: H = 11.96, *p* < 0.001, Additional file 6: Figure S2). However, there was no difference in CFU between all three bacterial groups (Kruskal-Wallis test for non-parametric data: H = 1.67, *p* < 0.43).

#### Gene expression

**Fig. 4:**
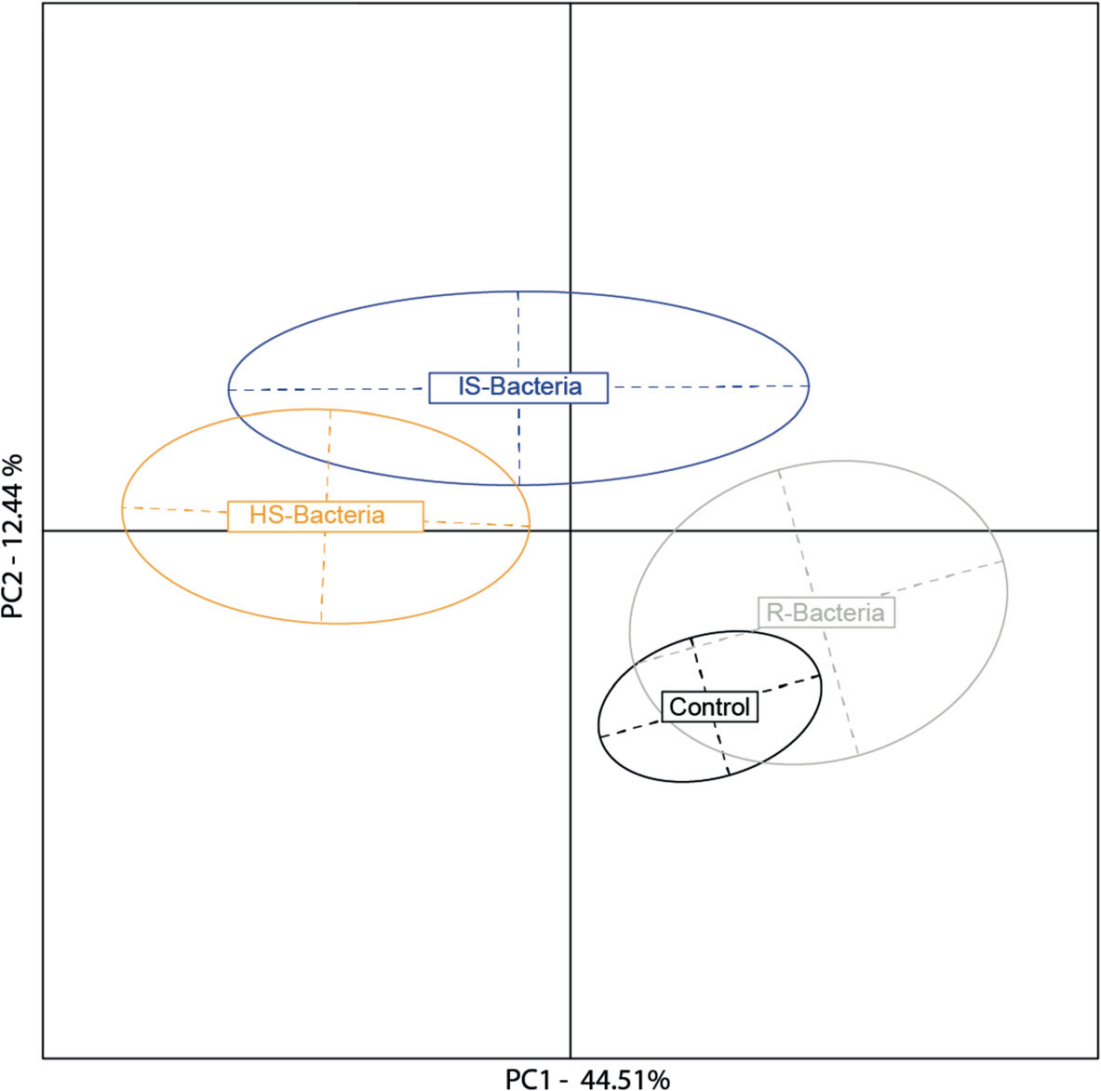
Ordination of differentially expressed immune-genes between four different infection treatments. Juvenile pipefish were either sham injected with PBS (*black*), infected with bacteria resistant to phage infection (*grey*), intermediate susceptible bacteria (*blue*), or highly susceptible bacteria (*orange*). Note: all experiments have been performed with strains of *Vibrio alginolyticus*

Bacterial group (HS, IS, R or control) significantly affected gene expression of infected juvenile pipefish, MANOVA (Pillai’s trace = 2.2, Approx. F_3_ = 1.62, *p* = 0.01). There was no difference in gene expression between sham-injected controls and pipefish infected with *Vibrio* strains resistant to phage infection (Fig. 4). However, gene expression differed significantly when pipefish were infected with *Vibrio* strains susceptible to phages. These observed differences in immune gene expression suggest that virulence on a eukaryotic host varies significantly between bacteria that have different phage-resistance phenotypes. Univariate ANOVAs revealed eleven genes that contribute to the observed significant group effect. Among these eleven genes, four genes belong to the innate and three to the adaptive immune system, while one gene belongs to the complement system and three genes are involved in gene silencing or deactivation (Additional file 7: Table S4).

### Genome screening

We found no differences in the structure of the CRISPR/Cas system among the complete sequenced *V. alginolyticus* strains. A comprehensive screening for virulence factors revealed the presence of a gene that encodes a zona occludens toxin (ZOT) like protein in each genome. No other virulence factors were found in the genomes. All *V. alginolyticus* strains display nearly complete modification of the GATC motif (m6A, underlined is the methylated base) as of 99%. Around 20% of all CCAGCANY (m4C) motifs were modified additionally. Low methylation fractions of strain K05K4 cannot be taken into account as the coverage requirements of 50% were not met.

### Bacterial properties

#### Growth rate

There was no difference in bacterial growth rate in Medium 101 over a 24 h period, linear mixed effect model, F_2, 6_ = 3.81, *p* = 0.09.

#### Lysis time

Lysis time varied significantly between bacteria, which contain prophages that differ in their infection profile, linear model, F_2_ = 7.5, *p* = 0.001. ‘Highly-infective’ phage lysates lysed their bacterial hosts on average after 81 min, while ‘intermediate-infective’ and ‘non-infective’ phage lysates took on average 105 and 100 min, respectively. Follow-up analysis revealed a significant difference in lysis time between bacteria that contain ‘highly-infective’ phage lysates and bacteria that contain ‘non-infective’ phage lysates t_62_ = -2.68, *p* = 0.025, as well as between ‘highly-infective’ phage lysates and ‘intermediate-infective’ phage lysates t_62_ = -3.86, *p* < 0.001. There was no significant difference in mean lysis time between ‘intermediate-infective’ and ‘non-infective’ phage lysates t_62_ = 0.21, *p* = 0.98.

#### Twitching motility

There was no significant difference in twitching motility between resistant and susceptible bacteria F_2, 63_ = 0.098, *p* = 0.91, indicating that pilus mutations, which could lead to reduced motility, were not the primary form of resistance against phages.

## Discussion

Virulence shifts through a hyperparasite can change dual species interactions with profound implications on ecosystem dynamics and human health [37]. We empirically investigated a tripartite host-parasite interaction focusing on two players each, namely phage infectivity against bacteria as well as bacterial virulence against a eukaryotic host and found evidence that both two-way interactions are linked. We could induce prophages from all bacterial isolates indicating that lysogeny is common in the genus *Vibrio*. We then determined *Vibrio* resistance to each of the phage lysates and tested the virulence of nine selected *Vibrio* strains against their final eukaryotic hosts. Our results suggest that phage-resistant strains are less harmful to their eukaryotic host than phage-susceptible strains. These findings indicate that bacteria with a phage susceptible phenotype are associated with higher virulence against eukaryotic hosts.

### Infectivity of phage lysates can be linked to bacterial resistance against superinfecting phages

The structure of phage-bacteria infection networks (PBINs) can range from random matrices over nearly diagonal matrices and nested structures to block-like matrices that exhibit high degrees of modularity [38, 39]. By generating a replicated 75 × 75 cross-infection matrix of *Vibrio* bacteria and phage lysates that were obtained from these bacteria by prophage induction, we found a clear-cut pattern between phage infection success and genetic distance of the host: *V. alginolyticus* genotypes were susceptible to phages from the same clade, but resistant to phages isolated from the *Splendidus* and the *Vulnificus* clade and vice versa*.* We are aware that the present PBIN comprises three different *Vibrio* clades with unequal sample sizes between clades and thus constrain the following discussion to the observed patterns within the *Alginolyticus* clade only.

Within the *Alginolyticus* clade we found a significantly nested structure (Fig. 1b). Nestedness results from sequences of gene-for-gene (GFG) coevolutionary adaptations and is the most common pattern in PBINs of natural communities [38, 39] but also in evolution experiments [40–42]. A nested structure results from cumulative GFG adaptations of bacterial resistance and phage infectivity, which confer resistance/infectivity against recently evolved phages/bacteria [39]. As a result, nested PBINs contain hierarchical interactions of phages and bacteria, which can be ordered according to the number of host genotypes/phage genotypes they can infect/resist. Likewise, according to their susceptibility to phages, bacteria from the present study can be grouped into three distinct categories: highly susceptible (HS), intermediate susceptible (IS) and resistant (R). This observed hierarchy indicates strong bacteria genotype by phage genotype interactions (GxG) and underlying GFG-like coevolutionary processes that characterize the present PBIN.

We found that 74 out of 75 bacterial isolates were immune to infection by their own lysate, indicating that homoimmunity is common for temperate filamentous *Vibriophages.* Indeed, most prophages immunize their host against their own kind and against phages of the same immunity group [4] for exceptions see [43]. We assume that a lytic infection in our spot assay is not possible if the superinfecting phage is homoimmune, i.e. it belongs to the same immunity group than the integrated prophage.

According to their infection pattern the 71 *alginolyticus* lysates could be grouped into 37 distinct groups, out of which 30 isolates had a unique infection profile. We further observed that most phage lysates isolated from HS-bacteria were non-infectious, while most phage lysates isolated from resistant bacteria could infect the majority of the tested bacteria isolates (Fig. 3). In addition, lysis time differed significantly between highly infective and non-infective as well as intermediate-infective phage lysates. Based on all these observed phenotypic properties we thus conclude that phage lysates of closely related host strains are different from each other.

Nevertheless, these phenotypic properties as well as the observed nestedness in the present infection matrix needs to be interpreted carefully by taking the potential multi-phage nature of the lysates into account. Whole genome sequencing of eight selected *Vibrio* strains indicates that resistant bacteria have more active prophages than susceptible bacteria (Fig. 2a). We could not detect a clear-cut pattern between bacterial-resistance phenotypes and the presence of particular phages, which are shared across genomes (Fig. 2a) nor across *Vibrio* phylogeny (Fig. 2b). It is thus tempting to speculate that resistance to phages and infectivity of the lysate correlates with the number of active prophages. In the first case we assume that more phages protect the bacterium from infection by additional phages, for instance by actively eliminating the infecting phage. In the latter case we predict that the probability to infect any given strain is higher the more active phages a lysate contains. If this holds true, the observed nested structure of the present PBIN may not be exclusively the result of classical GFG evolution between bacterial genotypes and phage genotypes (GxG) but rather a complex combination of underlying coevolutionary processes between lysogens (bacterial genomes plus integrated phage genomes) and phages [(G + G)xG].

The number of integrated prophages is not the sole factor that can influence bacterial resistance. Such an infection pattern could additionally be impacted by the presence of bacteriocins, e.g. colicin, which can also confer homoimmunity [44], the restriction modification system [45] or the involvement of the CRISPR/Cas system, which provides acquired immunity against mobile genetic elements by targeting invasive DNA in a sequence specific manner [46]. Based on the eight fully sequenced genomes we could not detect any differences in virulence factors, neither in the CRISPR/Cas system nor in the methylome of those strains. Mutations on specific cell surface components were assigned as an alternative mechanism explaining resistance to phages, for instance pili, which represent the main entry site for filamentous phages [47]. However, follow up experiments detected no difference in twitching motility between IS, HS and R bacteria, rejecting the hypothesis that R bacteria are resistant to superinfecting phages due to a pilus deficient mutant.

### Phage susceptible bacterial phenotypes may be associated with higher virulence against eukaryotic hosts

While it is acknowledged that prophages play an important role in bacterial virulence and evolution [48], the coupling between bacterial virulence against eukaryotic hosts and bacterial resistance against temperate phages has received little attention. Using a controlled infection experiment with selected strains that vary in their resistance to temperate phages, we tested whether bacterial resistance to phages and bacterial harm to eukaryotic hosts can be linked. While the amount of CFU in infected pipefish did not differ among treatment groups, host transcriptional response, notably expression of immune genes differed significantly between phage resistant and phage susceptible bacteria. We suggest that this observed difference in immune gene expression is linked to differences in virulence between phage resistant and phage susceptible strains. This indicates that the harm to the eukaryotic host and thus the virulence of a phage-resistant strain is significantly lower compared to the harm by a phage-susceptible strain.

The observed resistance-virulence trade-off has been frequently observed with lytic phages [6, 49, 50], for a recent review see [2] but has to our knowledge never been described for temperate phages. Common mechanisms/theories from studies using lytic phages explaining this trade-off in gram-negative bacteria are modifications of cell wall receptors, such as outer membrane proteins (OMPs) and Lipopolysaccharides (LPS) or bacterial appendices, such as flagellae or pili [2]. As known, filamentous phages enter the bacterium via the pilus [51], and no difference in twitching motility between phage susceptible and phage resistant strains could be detected, which would have suggested pilus-deficient mutants, the above mentioned mechanisms cannot explain the observed pattern. So far, we lack insight into the exact mechanism that couples virulence against eukaryotic hosts and resistance to temperate phages. The major difference between those closely related isolates is due to different prophages at different insertion sites, which can explain the distinct phenotypes. Thus we assume, that temperate phages are involved in mediating bacterial virulence and resistance.

There are different ways how prophages can contribute to the success of their bacterial hosts during infection. On the one side, prophages, and in particular filamentous phages are capable of influencing the virulence and evolution of their host by lysogenic conversion (for a recent review see [52]), with the most prominent example being the *Vibrio cholerae* CTXΦ phage carrying the cholera toxin gene [10]. However, in the case of prophages that do not contribute a clear phenotype such as virulence genes [53], their contribution to the fitness of the bacterial host is still unknown. In this context, we found that virulent strains (HS- and IS- bacteria) contain on average less active prophages than non-virulent strains (R-bacteria). In addition, the harm of selected strains did not depend on the presence of specific active prophages. Thus, our results suggest that (1) filamentous vibriophages do not always increase bacterial virulence but can also have opposite effects and (2) prophages may have more subtle effects on bacterial virulence apart from providing specific virulence toxins.

## Conclusion

Based on an empirical approach that goes beyond a classical dual host-parasite interaction, we show that phage-resistant bacteria strains harm their eukaryotic host less than phage-susceptible bacteria strains. These results illustrate the importance of hyperparasitism and that dual host-parasite interactions should not be studied in isolation. Ecological and evolutionary outcomes predicted by classical pairwise interactions differ profoundly, if we take additional players into account [54–56]. However, multiplayer interactions are only beginning to be explored [55], and are mostly limited to host-plant interactions as reviewed in [56], while studies using animal hosts are rare.

Phages are the most abundant entity in aquatic systems [57, 58] and their ecological importance in the marine environment has gained much attention in the last decade; for detailed reviews see [59–62]. Especially prophages have become recognized as important components of the marine environment through their ability to manipulate bacterial properties, such as pathogenicity. Our experimental results demonstrate that if we are to understand the spread and evolution of prophage-mediated diseases, it is paramount to take an integrative view across more than two levels by considering the interaction between all species involved.

## Additional files

Additional file 1: Table S1. MLSA Primer information. (DOCX 14 kb)

Additional file 2: Table S5. The number of postfiltered reads and the average read length of the reads. (DOCX 14 kb)

Additional file 3: Table S2. Fluidigm Primer information. (DOCX 20 kb)

Additional file 4: Table S3. Average nulceotide identity between complete sequenced *Vibrio alginolytcus* genomes. (DOCX 14 kb)

Additional file 5: Figure S1. Original sorted and nested sorted matrices of each replicate of the qualitative assays. Rows and columns represent bacteria and phages. A black square indicates an interaction, i.e. infection success as determined by plaque formation. White cells refer to no infection, i.e. absence of plaques. (PDF 4077 kb)

Additional file 6: Figure S2. Number of colony forming units in infected pipefish differentiated by bacterial group as well as non-infected pipefish (PBS control). (PDF 117 kb)

Additional file 7: Table S4. Univariate ANOVAs of each immune gene of pipefish infected with R-, IS-, and HS bacteria. Bacterial group was treated as a fixed factor and each single strain was nested in its bacterial group. Significant *p*-values are presented in boldface. (DOCX 34 kb)

## Abbreviations

CFU: Colony forming units
HI-, II-, NI-phages: Highly infective, intermediate infective, non infective phages
HS-, IS-, R-bacteria: High susceptible, intermediate susceptible, resistant bacteria
MLSA: Multi locus sequence analysis
PBN: Phage bacteria infection network
