# Supplementary material for: Tripartite species interaction: eukaryotic hosts suffer more from phage susceptible than from phage resistant bacteria
Source: BMC Evol Biol. 2017 Apr 11;17:98. doi: 10.1186/s12862-017-0930-2 (PMC5387238; doi:10.1186/s12862-017-0930-2)
Supplement: Supplementary file 1 — MLSA Primer information. (DOCX 14 kb) [file 12862_2017_930_MOESM1_ESM.docx]

Additional file 1: Table S1: MLSA Primer information

| Gene product | Primer name | Sequence (5'-3') |  |
| --- | --- | --- | --- |
| Uridylat Kinase | pyrH-02-R | GTRAABGCNGMYARRTCCA |  |
|  | pyrH-04_F | ATGASNACBAAYCCWAAACC |  |
| 16S rRNA | 16S 27-F | AGAGTTTGATCATGGCTCAG |  |
|  | 16S 1492-R | TACCTTGTTACGACTT |  |
| Recombinase A | recA 01 F | TGARAARCARTTYGGTAAAGG |  |
|  | recA 01 R | TCRCCNTTRTAGCTRTACC |  |
|  |  |  |  |
