# Supplementary material for: Tripartite species interaction: eukaryotic hosts suffer more from phage susceptible than from phage resistant bacteria
Source: BMC Evol Biol. 2017 Apr 11;17:98. doi: 10.1186/s12862-017-0930-2 (PMC5387238; doi:10.1186/s12862-017-0930-2)
Supplement: Supplementary file 2 — The number of postfiltered reads and the average read length of the reads. (DOCX 14 kb) [file 12862_2017_930_MOESM2_ESM.docx]

Additional file 2: Table S5: The number of postfiltered reads and the average read length of the reads

| ***Vibrio* *alginoliticus* Strain** | **Postfiltered reads** | **Average read length (bp)** | **Number of contigs** |
| --- | --- | --- | --- |
| K01M1 | 77,729 | 14,074 | 2 |
| K04M1 | 82,665 | 13,291 | 5 |
| K04M3 | 81,315 | 13,965 | 3 |
| K04M5 | 79,409 | 14,098 | 3 |
| K05K4 | 20,039 | 10,823 | 5 |
| K06K5 | 65,017 | 13,905 | 4 |
| K09K1 | 79,297 | 13,846 | 5 |
| K10K4 | 70,876 | 13,262 | 2 |
