# Supplementary material for: Tripartite species interaction: eukaryotic hosts suffer more from phage susceptible than from phage resistant bacteria
Source: BMC Evol Biol. 2017 Apr 11;17:98. doi: 10.1186/s12862-017-0930-2 (PMC5387238; doi:10.1186/s12862-017-0930-2)
Supplement: Supplementary file 3 — Fluidigm Primer information. (DOCX 20 kb) [file 12862_2017_930_MOESM3_ESM.docx]

Additional file 3: Table S2: Fluidigm Primer information

| **Gene** | **function** | **Primer: Forward (5' - 3')** | **Primer: Reverse (5' - 3')** | **length** | **Blastx** |
| --- | --- | --- | --- | --- | --- |
| lymph-antigen75 | adaptive | GCGCGGATATCCTAACCAT | CATGAGTGCCATCGTACCAC | 150 | XP_005724198.1 |
| HIVEP2 A | adaptive | CCATACAGGTGAAAGACCTTATCC | TTCTGTATGTTCCTCAACGTCTTC | 190 | XP_696438.4 |
| HIVEP3 | adaptive | CGCTGACACTCCAGGTAGAATAG | TTAACCTCTGTCAACTGGTGCTAC | 111 | XP_008321814.1 |
| lympcyt A | adaptive | CATACACACTCATGGTGCTTCAC | TGATAGTTGATGATGTCACAGACG | 136 | XP_007577143.1 |
| CD45 A | adaptive | CGTCACGTCAAGAGGAAAGAC | AATGGACCACGACTGGAGGA | 190 | ACI46145.1 |
| Integ-Bt A | adaptive | ACAAGGAGTGCGTCCAGTGT | GCGTAGGTGTAGTAGAACCAGCAG | 193 | XP_008305870.1 |
| IgM-lc A | adaptive | GTGACCCTCTTCCCTCCTTC | CACCTACATCAGCCTCTTCCAG | 166 | BAB91000.1 |
| c3 | complement | AGACCCCAACATGAAGCAGT | CTCTCTCAATAGGCTCCATGC | 191 | ADU33222.1 |
| C9 A | complement | CAGCCCATTTATACGCTGGT | AGCCATTCCCTCAAAGAGGT | 197 | AFU81223.1 |
| C1Q-sco A | complement | GTGATCATAGAGGTCGTCATTCAG | CTTCAAGATGAGTTGTCACATGGT | 150 | XP_008426360.1 |
| lectpII | innate | ATCCGCTCGAATCAGTTCA | AGGCCGTGTGCAACATTC | 90 | AAQ56012.1 |
| lectptI | innate | CGCCTCAATCAGTTCACGA | CGAGAGCGTCTGCAACATT | 91 | AAQ56013.1 |
| coagfactor2 | innate | TTACAGAGCGGCCTCACC | TCCAGATGCAAAAGCAGGTC | 176 | XP_006626829.1 |
| hsp1-60kda | innate | GTACGGTCATCATCGAGCAG | AGCACTGTGGCAGTGGTG | 152 | AAV40980.1 |
| ik-cytokine | innate | GCCGAGTGTTACCCTGCTAC | CAGCGCCTCCTTGTTGTT | 162 | XP_004557045.1 |
| IL-10 | innate | TTCCTGACTGCACAGTTGCT | TCTTCGATTGTCTGGTCGAG | 188 | XP_004069312.1 |
| kinesin-13b | innate | CGACAACAACCCAGACTCAG | TGTGGGTCTTTGGTGCTG | 226 | XP_005447761.1 |
| nramp | innate | GAGTGGTTCTGGGCTGTTTC | CAGTAGCACTCGTGCGAAAC | 164 | XP_009098790.1 |
| AIF-A | innate | GGCTTTTCTGACCGATGAAC | CTCCTCCTGCCACTTCTGAC | 204 | XP_007559540.1 |
| TSPO B | innate | TGGGGTATGGCTCCTATCTG | CCGGTTAGAAGCACGATCTC | 173 | ACQ58389.1 |
| LPS-TNF A | innate | GTAGAGTATAAGCCAGGCCTGAAG | AGTCGTCCACACAGAATGGTATC | 100 | ADZ99105.1 |
| tranfe A | innate | AATGCGCCACTGTAGTCATAGTAA | ATTAAGATGGACGTGCTGAAGTG | 182 | XP_006786562.1 |
| calrcul A | innate | TGCACGTCATCTTTAACTACAAGG | GCCAGACTCAACCTTCTTATTGTC | 176 | XP_004068493.1 |
| intf A | innate | ATCTTCTCCATCAAGGCCAGAG | GCATATACAAGGACCATAACGAAG | 144 | NP_001028804.1 |
| IL8 A | innate | GCGACTACAACCCACACTGC | GACAGACTCGTTCACCACTCAC | 172 | AGR27883.1 |
| TAP A | innate | GAGGACTCCATGGTGATGTTG | GCGGGTAAGTCTCTTCAATTCTC | 137 | XP_004572711.1 |
| Tyroprot | innate | GAACGACAGTTGCTCTCCACT | CTGAACCAGCTGTTGTAGTGAATC | 210 | XP_008283842.1 |
| CK7 | innate | TCAGATCATTGCCACCATAAAG | GGGCAGGTACAGATGTCTTGTT | 142 | ADE58987.1 |
| Lys1 | innate | CATACACACTCATGGTGCTTCAC | TGATAGTTGATGATGTCACAGACG | 136 | XP_007577143.1 |
| Jmjc-PhD | Lysine- demethylase | CACATTGGTTTGTGCGATAGA | CCAGACCTCCTCCATCAACTC | 88 | XP_008318334.1 |
| No66 | Lysine- demethylase | GAACTCCACATCCTCCTCCA | TACCGTCTCCTCCTACCAGA | 97 | XP_005935471.1 |
| TPR | Lysine- demethylase | GAAGTAGACCATTCCCAGACCA | AAGCATTATCGGCATACCAGAG | 92 | XP_006795785.1 |
| BROMO | Histone Acetylase | GAGCCAGTAGGTTCTTGAGCAT | AAGCAGAGTCAGATCAGGAAGG | 184 | XP_008283083.1 |
| MYST | Histone Acetylase | ATCCTCACCGAAGTCAACAAG | GCTCGTAACTGAAGGCGATG | 158 | AAI55278.1 |
| HDAC1 | Histone Deacetylase | GTAATCGCTGGTGTTCTGGTTG | CACGCTGTTGGACCTATGAAA | 149 | XP_004566614.1 |
| HDAC3 | Histone Deacetylase | CGTATGCTGAGGTTGAAGCA | CAGAGTTACAGGCAGCTATTCCA | 134 | XP_003977098.1 |
| HDAC6 | Histone Deacetylase | ATGTGAGTGAGGTGGGCATAG | CGAGTTCAATCCAAGTCTCGTT | 111 | XP_006810086.1 |
| ASH | Histone methyltransferase | CACTCTGGGCTTCCTCATTGT | CTCTTCTCGGCTTTGTCCAC | 145 | XP_003439681.1 |
| DNMt1-2 | DNA methyltransferase | CTTCAACTCTCGCACCTACTCA | AGTTCCTCACATTCTCCAGCA | 111 | XP_006631651.1 |
| DnMt3A | DNA methyltransferase | CCATTCGATCTGGTGATAGGAG | GTTCCCTCATAAAGACCCTTCC | 84 | XP_008932658.1 |
| DnMt3A-2 | DNA methyltransferase | GTGTGTGGATCTGTTGGTAGGA | CTCAAAGTCCTGTTCGTGATTG | 167 | XM_008279017 |
| DnMt3B | DNA methyltransferase | GTGCTTCTGGGATGGACAAG | GAGTTGGAGCGTGTGGTGAT | 101 | XP_003962984.1 |
| N6admet | DNA methyltransferase | AGATGAAGTCGTCGCCATAGAT | GCAACACGATTAGCTGAGGAG | 182 | NP_080802.1 |
| TAF8 | Transcriptionfactor | ACATTCCGTGAACCAGTCTCAG | ATCAATGGGAAAGAGGTGATGT | 152 | ACN10220.1 |
| G6PDH A | housekeeping | ATCTTCACTCCACTCCTTCATCA | GTAAGTGCCTTCATAGCGGAATC | 132 | XP_007566410.1 |
| ddpgly B | housekeeping | CTAGCCTCCTTGATGCACTGT | AGATGATGAGATGGTCGTACAGG | 199 | XP_008411280.1 |
| ribop B | housekeeping | AAGAGAAGAAGGAGGAGCAGGT | CACGAAGGTGTCGTTGAAAG | 107 | KFP09164.1 |
| UBI | housekeeping | CGTGAAGACATTGACGGGTA | GCAGCACCAGATGAAGAGTG | 196 | AEP96154.1 |
