# Supplementary material for: Tripartite species interaction: eukaryotic hosts suffer more from phage susceptible than from phage resistant bacteria
Source: BMC Evol Biol. 2017 Apr 11;17:98. doi: 10.1186/s12862-017-0930-2 (PMC5387238; doi:10.1186/s12862-017-0930-2)
Supplement: Supplementary file 4 — Average nulceotide identity between complete sequenced Vibrio alginolytcus genomes. (DOCX 14 kb) [file 12862_2017_930_MOESM4_ESM.docx]

Additional file 4: Table S3: Average nulceotide identity between complete sequenced *Vibrio alginolytcus* genomes

|  | K01M1 | K04M1 | K04M3 | K04M5 | K05K4 | K06K5 | K09K1 | K10K4 |
| --- | --- | --- | --- | --- | --- | --- | --- | --- |
| K01M1 | NA | 0.9996 | 0.9996 | 0.9996 | 0.9996 | 0.9996 | 0.9996 | 0.9996 |
| K04M1 |  | NA | 0.9996 | 0.9996 | 0.9996 | 0.9996 | 0.9996 | 0.9996 |
| K04M3 |  |  | NA | 0.9995 | 0.9995 | 0.9995 | 0.9997 | 0.9995 |
| K04M5 |  |  |  | NA | 0.9996 | 0.9995 | 0.9997 | 0.9995 |
| K05K4 |  |  |  |  | NA | 0.9996 | 0.9996 | 0.9996 |
| K06K5 |  |  |  |  |  | NA | 0.9996 | 0.9996 |
| K09K1 |  |  |  |  |  |  | NA | 0.9996 |
| K10K4 |  |  |  |  |  |  |  | NA |
