# Supplementary material for: Tripartite species interaction: eukaryotic hosts suffer more from phage susceptible than from phage resistant bacteria
Source: BMC Evol Biol. 2017 Apr 11;17:98. doi: 10.1186/s12862-017-0930-2 (PMC5387238; doi:10.1186/s12862-017-0930-2)
Supplement: Supplementary file 5 — Original sorted and nested sorted matrices of each replicate of the qualitative assays. Rows and columns represent bacteria and phages. A black square indicates an interaction, i.e. infection success as determined by plaque formation. White cells refer to no infection, i.e. absence of plaques. (PDF 4077 kb) [file 12862_2017_930_MOESM5_ESM.pdf]

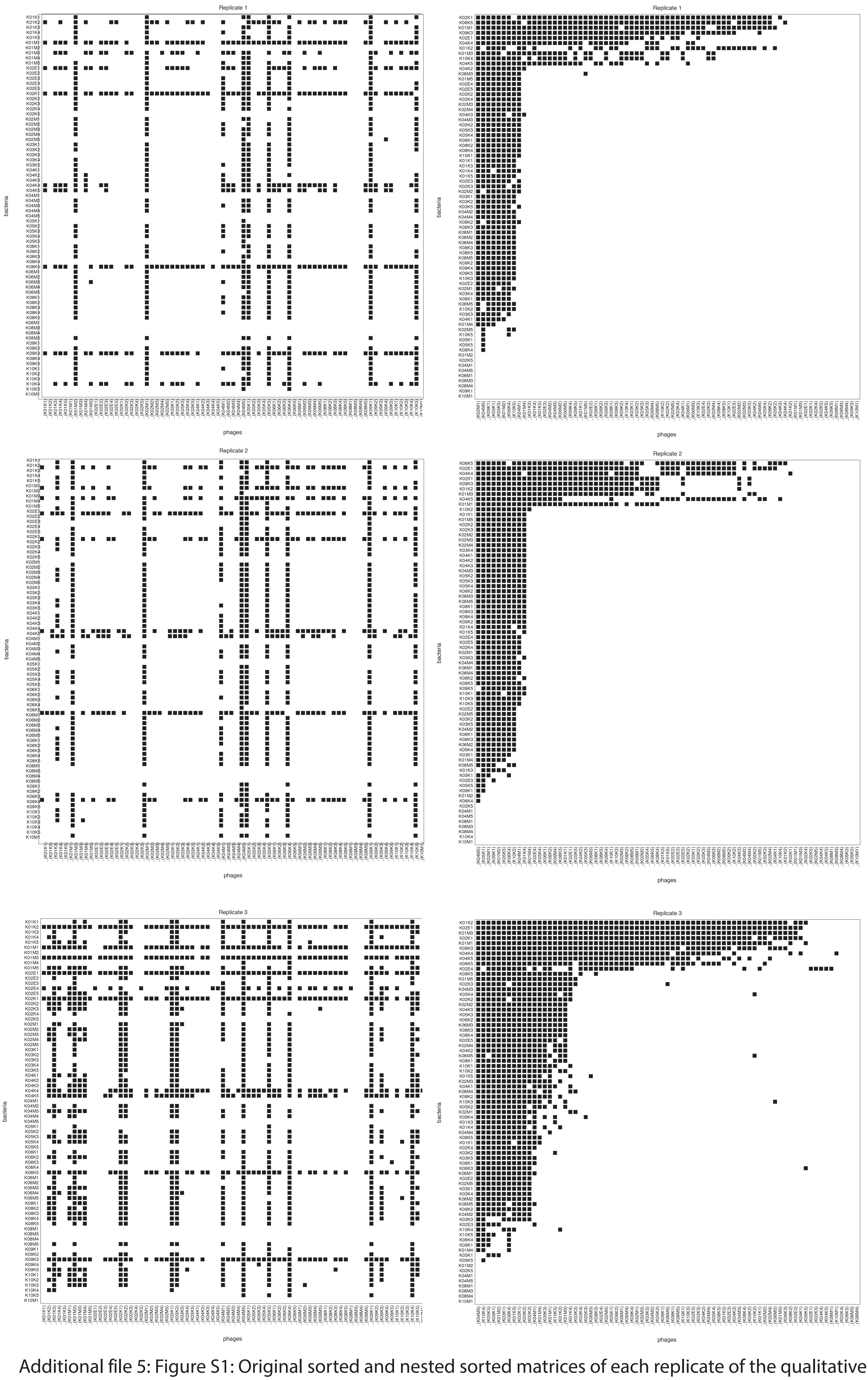

Additional file 5: Figure S1: Original sorted and nested sorted matrices of each replicate of the qualitative assays. Rows and columns represent bacteria and phages. A black square indicates an interaction, i.e. infection success as determined by plaque formation. White cells refer to no infection, i.e. absence of plaques.
