# Supplementary material for: Tripartite species interaction: eukaryotic hosts suffer more from phage susceptible than from phage resistant bacteria
Source: BMC Evol Biol. 2017 Apr 11;17:98. doi: 10.1186/s12862-017-0930-2 (PMC5387238; doi:10.1186/s12862-017-0930-2)
Supplement: Supplementary file 6 — Number of colony forming units in infected pipefish differentiated by bacterial group as well as non-infected pipefish (PBS control). (PDF 117 kb) [file 12862_2017_930_MOESM6_ESM.pdf]

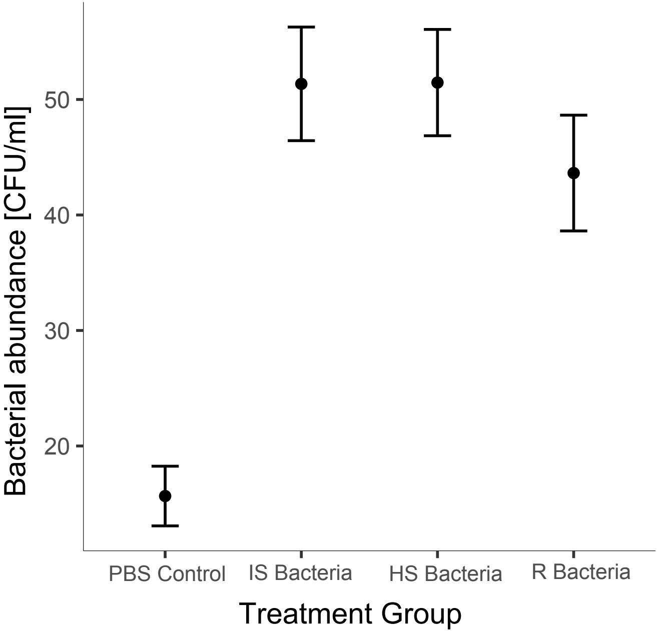

Additional file 6: Figure S2: Number of colony forming units in infected pipefish differentiated by bacterial group as well as non-infected pipefish (PBS control).
