# Supplementary material for: Tripartite species interaction: eukaryotic hosts suffer more from phage susceptible than from phage resistant bacteria
Source: BMC Evol Biol. 2017 Apr 11;17:98. doi: 10.1186/s12862-017-0930-2 (PMC5387238; doi:10.1186/s12862-017-0930-2)
Supplement: Supplementary file 7 — Univariate ANOVAs of each immune gene of pipefish infected with R-, IS-, and HS bacteria. Bacterial group was treated as a fixed factor and each single strain was nested in its bacterial group. Significant p-values are presented in boldface. (DOCX 34 kb) [file 12862_2017_930_MOESM7_ESM.docx]

Additional file 7: Table S4: Univariate ANOVAs of each immune gene of pipefish infected with R-, IS-, and HS bacteria. Bacterial group was treated as a fixed factor and each single strain was nested in its bacterial group. Significant p-values a presented in boldface.

| Gene | Fixed factor | Sum Sq | Mean Sq | F-value | p-value |
| --- | --- | --- | --- | --- | --- |
| ASH | Group | 0.19 | 0.06 | 0.37 | 0.78 |
|  | Group:Strain | 1.20 | 0.20 | 1.13 | 0.35 |
|  | Residuals | 11.13 | 0.18 |  |  |
|  |  |  |  |  |  |
| BROMO | Group | 0.61 | 0.20 | 0.84 | 0.48 |
|  | Group:Strain | 2.55 | 0.42 | 1.76 | 0.12 |
|  | Residuals | 15.18 | 0.24 |  |  |
|  |  |  |  |  |  |
| C1Q-scoA | Group | 0.20 | 0.07 | 0.27 | 0.85 |
|  | Group:Strain | 2.10 | 0.35 | 1.46 | 0.21 |
|  | Residuals | 15.16 | 0.24 |  |  |
|  |  |  |  |  |  |
| **c3** | **Group** | **15.87** | **5.29** | **2.91** | **0.04** |
|  | **Group:Strain** | **28.42** | **4.74** | **2.61** | **0.03** |
|  | Residuals | 114.53 | 1.82 |  |  |
|  |  |  |  |  |  |
| C9A | Group | 10.70 | 3.57 | 2.52 | 0.07 |
|  | Group:Strain | 18.07 | 3.01 | 2.13 | 0.06 |
|  | Residuals | 89.28 | 1.42 |  |  |
|  |  |  |  |  |  |
| calrcul_A | Group | 1.29 | 0.43 | 0.78 | 0.51 |
|  | Group:Strain | 3.49 | 0.58 | 1.05 | 0.40 |
|  | Residuals | 34.87 | 0.55 |  |  |
|  |  |  |  |  |  |
| **CD_45_A** | **Group** | **6.65** | **2.22** | **2.84** | **0.05** |
|  | Group:Strain | 5.54 | 0.92 | 1.18 | 0.33 |
|  | Residuals | 49.21 | 0.78 |  |  |
|  |  |  |  |  |  |
| CK7 | Group | 2.18 | 0.73 | 0.61 | 0.61 |
|  | Group:Strain | 9.55 | 1.59 | 1.34 | 0.25 |
|  | Residuals | 74.81 | 1.19 |  |  |
|  |  |  |  |  |  |
| coagfactor2 | Group | 1.28 | 0.43 | 1.12 | 0.35 |
|  | Group:Strain | 0.32 | 0.05 | 0.14 | 0.99 |
|  | Residuals | 23.99 | 0.38 |  |  |
|  |  |  |  |  |  |
| DNMt1_2 | Group | 1.37 | 0.46 | 2.00 | 0.12 |
|  | Group:Strain | 1.86 | 0.31 | 1.36 | 0.25 |
|  | Residuals | 14.33 | 0.23 |  |  |
|  |  |  |  |  |  |
| **DnMt3A** | **Group** | **4.89** | **1.63** | **5.15** | **<0.001** |
|  | **Group:Strain** | **7.05** | **1.18** | **3.71** | **<0.001** |
|  | Residuals | 19.94 | 0.32 |  |  |
|  |  |  |  |  |  |
| **DnMt3A_2** | **Group** | **3.40** | **1.13** | **2.92** | **0.04** |
|  | **Group:Strain** | **11.34** | **1.89** | **4.86** | **<0.001** |
|  | Residuals | 24.47 | 0.39 |  |  |
|  |  |  |  |  |  |
| DnMt3B | Group | 2.00 | 0.67 | 2.07 | 0.11 |
|  | Group:Strain | 2.61 | 0.44 | 1.35 | 0.25 |
|  | Residuals | 20.28 | 0.32 |  |  |
|  |  |  |  |  |  |
| HDAC1 | Group | 0.83 | 0.28 | 1.12 | 0.35 |
|  | Group:Strain | 2.60 | 0.43 | 1.74 | 0.13 |
|  | Residuals | 15.70 | 0.25 |  |  |
|  |  |  |  |  |  |
| HDAC3 | Group | 0.61 | 0.20 | 0.83 | 0.49 |
|  | Group:Strain | 2.13 | 0.36 | 1.45 | 0.21 |
|  | Residuals | 15.49 | 0.25 |  |  |
|  |  |  |  |  |  |
| HDAC6 | Group | 2.29 | 0.76 | 2.51 | 0.07 |
|  | Group:Strain | 2.90 | 0.48 | 1.59 | 0.16 |
|  | Residuals | 19.16 | 0.30 |  |  |
|  |  |  |  |  |  |
| HIVEP2_A | Group | 3.65 | 1.22 | 2.19 | 0.10 |
|  | Group:Strain | 19.63 | 3.27 | 5.88 | <0.001 |
|  | Residuals | 35.05 | 0.56 |  |  |
|  |  |  |  |  |  |
| **HIVEP3** | **Group** | **4.13** | **1.38** | **3.84** | **0.01** |
|  | **Group:Strain** | **8.18** | **1.36** | **3.80** | **<0.001** |
|  | Residuals | 22.59 | 0.36 |  |  |
|  |  |  |  |  |  |
| hsp1_60kda | Group | 1.34 | 0.45 | 1.27 | 0.29 |
|  | Group:Strain | 1.38 | 0.23 | 0.66 | 0.68 |
|  | Residuals | 22.12 | 0.35 |  |  |
|  |  |  |  |  |  |
| IgM_lc_A | Group | 0.51 | 0.17 | 0.29 | 0.83 |
|  | Group:Strain | 3.20 | 0.53 | 0.91 | 0.49 |
|  | Residuals | 36.87 | 0.59 |  |  |
|  |  |  |  |  |  |
| ik_cytokine | Group | 0.65 | 0.22 | 0.74 | 0.53 |
|  | Group:Strain | 2.53 | 0.42 | 1.44 | 0.21 |
|  | Residuals | 18.46 | 0.29 |  |  |
|  |  |  |  |  |  |
| IL_10 | Group | 3.00 | 1.00 | 0.51 | 0.68 |
|  | Group:Strain | 10.15 | 1.69 | 0.86 | 0.53 |
|  | Residuals | 124.45 | 1.98 |  |  |
|  |  |  |  |  |  |
| IL8_A | Group | 0.07 | 0.02 | 0.02 | 1.00 |
|  | Group:Strain | 6.07 | 1.01 | 0.98 | 0.45 |
|  | Residuals | 65.27 | 1.04 |  |  |
|  |  |  |  |  |  |
| Integ_Bt_A | Group | 0.61 | 0.20 | 0.44 | 0.72 |
|  | Group:Strain | 2.35 | 0.39 | 0.85 | 0.53 |
|  | Residuals | 28.88 | 0.46 |  |  |
|  |  |  |  |  |  |
| intf_A | Group | 1.13 | 0.38 | 0.99 | 0.40 |
|  | Group:Strain | 4.17 | 0.69 | 1.83 | 0.11 |
|  | Residuals | 23.97 | 0.38 |  |  |
|  |  |  |  |  |  |
| **Jmjc_PhD** | **Group** | **6.57** | **2.19** | **5.39** | **<0.001** |
|  | **Group:Strain** | **9.09** | **1.51** | **3.73** | **<0.001** |
|  | Residuals | 25.59 | 0.41 |  |  |
|  |  |  |  |  |  |
| lectpII | Group | 1.89 | 0.63 | 0.80 | 0.50 |
|  | Group:Strain | 9.94 | 1.66 | 2.11 | 0.06 |
|  | Residuals | 49.55 | 0.79 |  |  |
|  |  |  |  |  |  |
| lectptI | Group | 0.43 | 0.14 | 0.12 | 0.95 |
|  | Group:Strain | 8.01 | 1.34 | 1.15 | 0.35 |
|  | Residuals | 73.42 | 1.17 |  |  |
|  |  |  |  |  |  |
| LPS_TNF_A | Group | 3.28 | 1.09 | 0.88 | 0.46 |
|  | Group:Strain | 19.28 | 3.21 | 2.59 | 0.03 |
|  | Residuals | 78.21 | 1.24 |  |  |
|  |  |  |  |  |  |
| lympcyt_A | Group | 1.74 | 0.58 | 1.93 | 0.13 |
|  | Group:Strain | 2.76 | 0.46 | 1.53 | 0.18 |
|  | Residuals | 18.96 | 0.30 |  |  |
|  |  |  |  |  |  |
| **lymph_antigen75** | **Group** | **5.57** | **1.86** | **3.69** | **0.02** |
|  | **Group:Strain** | **10.09** | **1.68** | **3.34** | **0.01** |
|  | Residuals | 31.69 | 0.50 |  |  |
|  |  |  |  |  |  |
| Lys1 | Group | 4.72 | 1.57 | 1.26 | 0.29 |
|  | Group:Strain | 4.04 | 0.67 | 0.54 | 0.77 |
|  | Residuals | 78.38 | 1.24 |  |  |
|  |  |  |  |  |  |
| MYST | Group | 0.63 | 0.21 | 1.17 | 0.33 |
|  | Group:Strain | 1.36 | 0.23 | 1.27 | 0.29 |
|  | Residuals | 11.24 | 0.18 |  |  |
|  |  |  |  |  |  |
| N6admet | Group | 0.69 | 0.23 | 0.71 | 0.55 |
|  | Group:Strain | 1.14 | 0.19 | 0.58 | 0.75 |
|  | Residuals | 20.64 | 0.33 |  |  |
|  |  |  |  |  |  |
| No66 | Group | 2.00 | 0.67 | 2.08 | 0.11 |
|  | Group:Strain | 2.63 | 0.44 | 1.37 | 0.24 |
|  | Residuals | 20.13 | 0.32 |  |  |
|  |  |  |  |  |  |
| nramp | Group | 0.12 | 0.04 | 0.17 | 0.92 |
|  | Group:Strain | 0.74 | 0.12 | 0.54 | 0.78 |
|  | Residuals | 14.48 | 0.23 |  |  |
|  |  |  |  |  |  |
| **TAF8** | **Group** | **2.72** | **0.91** | **3.16** | **0.03** |
|  | Group:Strain | 3.37 | 0.56 | 1.95 | 0.09 |
|  | Residuals | 18.11 | 0.29 |  |  |
|  |  |  |  |  |  |
| TAP_A | Group | 0.92 | 0.31 | 0.97 | 0.41 |
|  | Group:Strain | 2.41 | 0.40 | 1.27 | 0.28 |
|  | Residuals | 19.99 | 0.32 |  |  |
|  |  |  |  |  |  |
| **TPR** | **Group** | **4.66** | **1.55** | **5.56** | **<0.001** |
|  | **Group:Strain** | **6.29** | **1.05** | **3.75** | **<0.001** |
|  | Residuals | 17.62 | 0.28 |  |  |
|  |  |  |  |  |  |
| tranfe_A | Group | 1.00 | 0.33 | 0.39 | 0.76 |
|  | Group:Strain | 9.72 | 1.62 | 1.90 | 0.09 |
|  | Residuals | 53.64 | 0.85 |  |  |
|  |  |  |  |  |  |
| TSPO_B | Group | 0.01 | 0.00 | 0.01 | 1.00 |
|  | Group:Strain | 0.60 | 0.10 | 0.31 | 0.93 |
|  | Residuals | 20.41 | 0.32 |  |  |
|  |  |  |  |  |  |
| **Tyroprot** | **Group** | **2.94** | **0.98** | **2.52** | **0.07** |
|  | **Group:Strain** | **12.63** | **2.11** | **5.40** | **<0.001** |
|  | Residuals | 24.58 | 0.39 |  |  |
|  |  |  |  |  |  |
